# Postpartum women’s prospective acceptability of long-acting HIV prevention approaches in Kenya: A qualitative study

**DOI:** 10.21203/rs.3.rs-6058755/v1

**Published:** 2025-02-27

**Authors:** Tessa Concepcion, John Kinuthia, Felix A Otieno, Eunita Akim, Helen Aketch, Laurén Gómez, Grace John-Stewart, Bih Moki Suh, Emmaculate M Nzove, Nancy Ngumbau, Jerusha N Mogaka, Sarah Obatsa, Ben O Odhiambo, Caroline Omom, Marin Strong, Anjuli D Wagner, Salphine Watoyi, Jillian Pintye

**Affiliations:** University of Washington; Kenyatta National Hospital; Kenyatta National Hospital; Kenyatta National Hospital; Kenyatta National Hospital; University of Washington; University of Washington; University of Washington; Kenyatta National Hospital; Kenyatta National Hospital; University of Washington; Kenyatta National Hospital; Kenyatta National Hospital; Kenyatta National Hospital; University of Washington; University of Washington; Kenyatta National Hospital; University of Washington

**Keywords:** Long-acting PrEP, maternal and child health, pregnancy, postpartum, acceptability, implementation strategies

## Abstract

**Background:**

New long-acting pre-exposure prophylaxis (LA-PrEP) options offer an alternative to daily oral PrEP, which poses difficulties for adherence, especially during pregnancy and postpartum. Yet, limited data exist on LA-PrEP acceptability among pregnant and postpartum women. We aimed to evaluate its acceptability and identify strategies to enhance it.

**Methods:**

We conducted an exploratory qualitative study with postpartum women in five public health facilities in Kisumu and Siaya Counties, Kenya. In-depth interviews (IDIs) were conducted with women expressing high, low, and mixed LA-PrEP interest throughout pregnancy and postpartum. Inductive and deductive content analysis was used, and themes of acceptability were explored using the Theoretical Framework of Acceptability (TFA).

**Results:**

We conducted 70 IDIs with postpartum women between August 2023 and March 2024. The majority (62.9%) expressed consistently high interest in LA-PrEP. Most viewed LA-PrEP, especially every two-month injectables, as highly acceptable due to reduced pill burden, side effects, and dosing frequency. Concerns were raised regarding injectable PrEP safety for the baby during pregnancy and suitability of using the vaginal ring during delivery. Participants emphasized the importance of education on the safety of these methods during pregnancy and breastfeeding, and strategies for improving adherence, such as mobile reminders. Overall, women preferred LA-PrEP options over daily oral PrEP for convenience, effectiveness, and privacy, with healthcare provider education seen as crucial.

**Discussion:**

We found high acceptability of LA-PrEP options among postpartum women with experience taking PrEP during pregnancy. The findings reveal diverse preferences and key factors influencing acceptability, including safety, discretion, and convenience.

## Introduction

In Eastern and Southern Africa, women experience a two-fold higher risk of HIV infection during pregnancy and postpartum compared to non-pregnant periods, and acute maternal infections substantially increase risk of vertical HIV transmission [1–3]. The World Health Organization (WHO) recommends safe and effective HIV prevention methods among populations at substantial risk of HIV, including tenofovir disoproxil fumarate (TDF)-based oral pre-exposure prophylaxis (oral PrEP), and long-acting (LA-) PrEP methods, such as the dapivirine vaginal ring (DVR) and long-acting intramuscular injectable cabotegravir (CAB-LA) [4–6]. Existing safety data on the use of DVR and CAB-LA use in pregnancy suggest favorable safety profiles [7, 8] and ongoing safety studies will provide more evidence to inform guidelines for the use of these prevention products in pregnancy.

Oral PrEP has robust evidence supporting its safety, efficacy, and acceptability during pregnancy and postpartum periods. However, pregnant and postpartum women often face challenges with adherence and persistence, often due to co-occurring side effects with pregnancy and the demands of motherhood [9–11]. LA-PrEP methods, such as DVR and CAB-LA, may alleviate some of these burdens [12–14]. Quantitative studies of LA-PrEP preferences among pregnant and breastfeeding women highlight the importance of offering a long-acting method with strong effectiveness and safety during pregnancy and breastfeeding, and an option that is free of charge [12]. Qualitative evaluations of LA-PrEP acceptability among pregnant and breastfeeding women are limited to DVR, highlighting both concerns about intravaginal use and benefits of discreet usage and a long-acting regimen [15]. Understanding acceptability of various PrEP methods from an end-user perspective is important to inform implementation and scale-up strategies [16–18]. Specifically, components of acceptability can inform PrEP counselling messaging, adherence support, and use of PrEP materials tailored to pregnancy and breastfeeding [19]. Despite this, there is limited qualitative evidence of pregnant and postpartum women’s perspectives on the relative acceptability for oral, vaginal ring, and injectable PrEP modalities.

The Theoretical Framework of Acceptability (TFA) was designed to support the assessment of acceptability of healthcare interventions within the development, piloting and feasibility, outcome and process evaluation, and implementation phases [14]. In this paper, we applied the TFA to in-depth interviews (IDIs) conducted among postpartum women who initiated daily oral PrEP during pregnancy. Our objective was to evaluate acceptability of LA-PrEP methods and identify strategies to enhance acceptability for pregnant and postpartum women.

## Methods

### Study design, setting and population

We conducted an exploratory qualitative study using content analysis among postpartum women receiving mobile PrEP-adherence support in the mWACh PrEP trial (ClinicalTrials.gov: NCT04472884). The trial took place in five public health facilities within Kisumu and Siaya Counties in Kenya. Participating facilities enrolled 600 pregnant women who were HIV negative, between 24 and 32 weeks of gestational age, and received antenatal at the facility. Women had monthly study visits during pregnancy and postpartum visits at six weeks, 14 weeks, six months, and nine months. Detailed descriptions of the trial design, methods, and findings are published elsewhere [20]. We purposively selected 70 participants who indicated having consistently high, consistently low, or mixed interest in LA-PrEP to participate in a single interview in order to elucidate varying perspectives of acceptability. IDIs were conducted after participants completed all clinical trial study visits.

### Data collection

All trial participants completed nurse administered quantitative questionnaires at each study visit that assessed sexual behavior; infant outcomes; and PrEP attitudes, use, and adherence. At enrollment, participants were asked about their demographic and social factors, such as household characteristics and adverse childhood experiences (ACE) [21]. Participants were informed about PrEP products that may be available to them in the future, including CAB-LA and DVR. At each study visit, participants were also asked “*A new form of PrEP might be available soon, which is an injection (e.g., shot) you would have to take every 8 weeks. How interested would you be in this form of PrEP delivery*?” Consistent high interest in LA-PrEP was defined as responding with “Very interested” or “Interested” at least once and never responding with “Neutral”, “Slightly interested”, or “Not interested” at any study visit. Consistent low interest in LA-PrEP was defined as responding with “Not interested” at least once and never responding with “Slightly interested”, “Neutral”, “Interested”, or “Very interested” at any study visit. Mixed interest was defined as any combination of responses not classified as “consistently high” or “consistently low”. We used these data to purposively select women with consistent high, consistent low, and mixed interest in LA-PrEP.

Interviewers (SO, HA, CO) attended a 3-day training session in July of 2023 and internally piloted materials. They were from the same region as participants, were female, held a bachelor’s degree, and have prior training and current employment in qualitative interviewing. Interviewers used a semi-structured interview guide with a section devoted to themes around LA-PrEP acceptability, informed by the TFA [14]. The interview guide was developed for this study and was a subset of the full interview guide used for the qualitative component of the mWACh PrEP trial (Supplemental file 1). A study staff member familiar with the selected participants contacted them by phone and introduced them to the interviewer, who then invited them to participate. During the interview, participants received a refresher on potential future LA-PrEP formulations. All IDIs were conducted in person at the clinic where the participant sought their perinatal care in a private room with no one else present. IDIs were conducted in the participants preferred language (English, Luo, or Swahili) and lasted approximately 60–90 minutes.

### Data management and analysis

IDIs were audio-recorded, transcribed, and translated into English. Interviewers completed debrief reports within 24 hours of completing an IDI. Ten percent of IDIs were back translated for quality assurance. Three authors reviewed transcripts for accuracy (EA, JN, SW). Two authors (JN and EA) developed an initial codebook and six authors (EA, TC, HA, SO, BMS, MS) refined the codebook using an inductive-deductive thematic content analysis approach [22]. First, all transcripts were reviewed, and a preliminary codebook was developed based on interview guides. Three transcripts were group-coded, and new themes were inductively added to the codebook. Codes and themes were continuously reviewed and revised, with discrepancies between coders resolved during weekly meetings until an agreement was reached. Once consensus was achieved, the remaining transcripts were divided among three authors (EA, SO, HA) for primary coding. Another three authors (TC, MS, BMS) secondary coded all transcripts to ensure consistency and accuracy. Coding and codebook refinement was conducted in Dedoose (Version 9.2.22, Los Angeles, CA). After coding was completed, key themes were summarized, and themes were organized into the domains of the TFA (Table 1). Implementation strategies to address specific areas of acceptability and illustrative quotes were also identified.

We acknowledge that our experiences, education, and positions may have contributed to data collection, interpretation, and presentation of our study findings. Our team included individuals with training in HIV counseling and testing, quantitative and qualitative methods, and socio-behavioral research to minimize bias and ensure culturally appropriate interpretation. At least one author of the same gender and geographic area as the participants was included in each step of data analysis to ensure appropriate interpretation of results. The consolidated criteria for reporting qualitative studies (COREQ) was used as a guide throughout data collection and analysis [23].

#### Ethical approvals

The University of Washington and Kenyatta National Hospital institutional review boards approved this study. All participants provided informed consent and consented to be audio recorded.

## Results

We conducted 70 IDIs with postpartum women between August 2023 and March 2024. All purposively sampled participants consented to participate in the study and none dropped out. A total of 44 participants (62.9%) had high consistently LA-PrEP interest throughout pregnancy and postpartum, one participant (1.4%) had consistently low LA-PrEP interest, and 25 participants (35.7%) had mixed LA-PrEP interest. Participants were approximately one year postpartum when interviewed (Median: 12.3 months, interquartile range [IQR]: 11.3–13.7 months). Median age at study enrollment was 25 years old (IQR: 22–30.5), most (n = 49, 70.0%) were currently married and had a secondary education or higher (n = 46,65.7%) (Table 2). A total of 44.3% (n = 31) had discontinued PrEP prior to the end of the study. About one-third were primigravida (n = 26, 37.1%).

**Figure 1:**
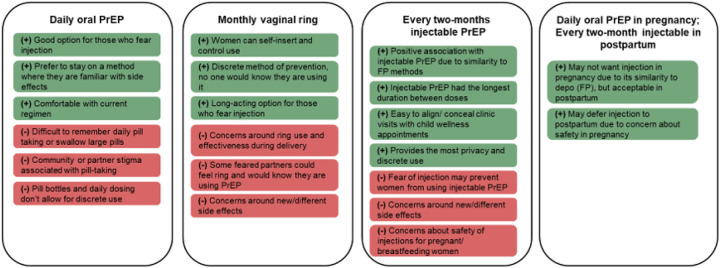
Participant’s perceived acceptability of PrEP options, stratifying positive (green) and negative (red) perceptions

Overall, most participants thought that LA-PrEP methods would be highly acceptable for themselves and pregnant/postpartum women in their community, with especially high acceptability identified for every two-month injectable PrEP. Figure 1 summarizes key themes identified and demonstrates how they fit within distinct profiles of preferred PrEP option as guided by domains of the TFA model.

### Acceptability themes related to pregnancy and postpartum periods

Safety for their pregnancy and baby was a top priority for participants, regardless of PrEP method or its effectiveness in preventing HIV acquisition. During pregnancy, some women preferred oral PrEP due to its familiarity and perceived safety for the baby and would defer taking injectable PrEP until after delivery because its unfamiliarity and limited availability of safety data (TFA domain: **Affective attitude**).

“… the injectable one, they are still doing research, the research is not complete. Maybe for the safety of the baby, if you are not pregnant it is okay, you can use it, like for me now I can use that injectable because I am not pregnant but because I have used the daily pill, the effects were not there, there were no effect on my baby, so I prefer maybe the pregnant women to just use the daily pill. Yeah, but if you are not pregnant, you can use the vaginal ring and the injectable”

(31 years, stopped PrEP in postpartum, high LA PrEP interest)

Despite familiarity with daily oral PrEP, adherence was seen as a burden in part due to competing household priorities and co-occurring side effects which could be exacerbated in pregnancy (TFA domain: **Burden**).

“Taking pills while you are pregnant is very hard, there are women who are given even the folic acid tablets to take and they don’t take it due to nausea”

(27 years, continued on PrEP, high LA PrEP interest)

However, routinizing follow-up with adherence counseling was seen as an opportunity to reinforce adherence messaging and support women’s PrEP continuation, especially during pregnancy.

“There are no issues of forgetting, especially because forgetting is an issue with pregnant mothers, you just need to remember the TCA date [Treatment Continuity and Adherence] after a while”

(38 years, continued on PrEP, mixed LA PrEP interest)

Many women believed that an injectable PrEP was the most effective option for HIV prevention, as it minimized opportunities for missed doses and ensured consistent coverage. In contrast, several participants had concerns about DVR’s ability to function in certain scenarios, such as delivery (TFA domain: **Perceived effectiveness**). They noted that PrEP choice requires comprehensive information about the side effects and safety of each method. They noted that women should be educated on how PrEP (all forms) can protect (and will not harm) their babies in pregnancy and breastfeeding (TFA domain: **Ethicality**).

“To the breastfeeding mothers I know most of them might be afraid taking PrEP… She might have refused taking the PrEP thinking it will affect [her] baby, so they should be informed.”

(22 years, stopped PrEP in pregnancy, high LA PrEP interest)

However, some participants voiced concerns about injectable PrEP, such as safety during pregnancy. Several women were also uneasy about DVR’s use during pregnancy and had concerns around the ring being in the vagina during delivery (TFA domain: **Intervention coherence**).

“Something that will make me fear is that it is injected direct in the blood, what if it can cause some illnesses, or if it can affect pregnant women and probably affect the unborn child.

(24 years, stopped PrEP in postpartum, high LA PrEP interest)

“Because with injectable, you will just get an injection but with this one, it is inserted. What about when you are due for delivery, it will still be inside the vagina.”

(31 years, continued on PrEP, mixed LA PrEP interest)

Women were confident in their ability to manage injectable PrEP, relating it to family planning (FP) injections. They noted that they could align injection visits with clinic visits for their pregnancy or baby, facilitating privacy and confidentiality. Some women thought DVR would be better for postpartum women as they can manage it themselves (TFA domain: **Self-efficacy**).

“If I get injected, who will know that I have been injected? Nobody, only me, yes. When I come to clinic, he [husband] will know that I have taken a baby to clinic”

(27 years, stopped PrEP in pregnancy, high LA PrEP interest)

“Pregnant women will prefer the injectable or the oral and the postpartum women will prefer the ring. Because I feel that there are women who just prefer the ring because they know when to replace it, and there are some who fear the injection and others also fear swallowing PrEP.”

(33 years, stopped PrEP in postpartum, high LA PrEP interest)

### LA-PrEP acceptability beyond pregnancy and postpartum

Participants also endorsed themes of acceptability that were not unique to pregnancy and postpartum periods. They expressed strong preferences for long-acting injectable PrEP due to its simplicity, convenience, and non-intravaginal use, favoring it over the DVR, which had mixed reactions. While some appreciated DVR for its long duration and non-injectable nature, others disliked it due to discomfort, fears of insertion, or social stigma: *“The ring is a no for me. Some people say it can get lost inside your vagina.” (38 years, continued on PrEP, mixed LA PrEP interest)*. However, both long-acting methods were considered easier to manage than daily oral PrEP due to reduced adherence burden.

“I would prefer to use it because it is just inserted and removed. Another reason why I like the ring more than the injectable, you know when you are injected it will last for two months, but the ring also lasts for 28 days, they are still all long term. I prefer the ring because I will not be injected.”

(32 years, stopped PrEP in postpartum, high LA PrEP interest)

Some women were concerned about forgetting injection dates, while others liked the routine nature of daily pills.

“As for the injection … also you can forget, let’s say after two months, you can forget then you say … ah this date expired yesterday… But you see, about the oral and you are seeing the bottle there, you will say let me go before this drug are over”

(18 years, stopped PrEP in postpartum, mixed LA PrEP interest)

Privacy was important to women, with injectable PrEP seen as more private and socially acceptable than oral PrEP.

“But personally, I would prefer the injectable because that one is discreet, no one will know about it, it shall be your own secret, and maybe together with the clinician who has injected you. This cannot cause a lot of family strife in the home.”

(32 years, stopped PrEP in pregnancy, mixed LA PrEP interest)

Women emphasized the importance of providing choices and information to make informed decisions and that they could choose the PrEP form that worked best for them.

“If I find that injection is good, it doesn’t cause me dizziness and backache, and doesn’t cause me any problem then I would continue with it. But in case it is causing me problems, then it will make me go back to the one that I was swallowing, because with it I don’t have much problem.”

(27 years, continued on PrEP, high LA PrEP interest)

### Strategies to support acceptability

Women recommended several strategies to support acceptability of various PrEP options in pregnant and postpartum periods. They emphasized that healthcare workers played an important role in educating pregnant and postpartum patients about PrEP options, side effects, safety, and effectiveness. They highlighted the value of information coming from doctors or healthcare professionals, stating “*we always believe what the doctors tell us, so as long as you assure me of its safety, I’ll be okay to use it.” (28 years, stopped PrEP in postpartum, mixed LA PrEP interest)*

Several women mentioned that education on how the vaginal ring works is particularly important for postpartum women as it could be misunderstood that it prevents pregnancy. Women emphasized the need for detailed information and education on how each method would affect their health, the potential side effects, and the proper way to use them.

“Later she might be regretting of her problem or the health care worker. That is why I said it is good for them to have a basic information such they know that the vaginal ring is only for preventing HIV and not pregnancy. Also other question can be if I am using it, can I use other forms of family planning, like coil?”

(20 years, continued on PrEP, high LA PrEP interest)

“Yes, you need to be open and tell us about the side effects of all these PrEP. For example, when I had the injection with the side effects, I may withdraw. But if we are told, we will stick to it as we allow the body to get used to it.”

(35 years, continued on PrEP, mixed LA PrEP interest)

Many women mentioned how mobile adherence support tools could be adapted to include LA-PrEP methods. specific aspects of adherence support included reminding users about injection dates, addressing concerns promptly, and providing education about side effects. Some participants suggested the introduction of visit reminders using SMS systems or aligning injection timepoints with pregnancy and postpartum visits.

## Discussion

We identified nuanced aspects of acceptance for these PrEP approaches in pregnant and postpartum periods, highlighting the heterogenous preferences and diverse needs of the population. Long-acting options were broadly accepted and perceived to alleviate the burden of daily pill taking. Injectable PrEP Specifically was valued for its discretion, alignment with familiar family planning methods, and long-duration. Despite the broad acceptance of new LA-PrEP methods, participants identified several themes specific to pregnancy and the postpartum period that could influence their acceptability. Concerns were raised about the safety of these products for their babies and the potential side effects during pregnancy, highlighting the need for robust safety data and comprehensive PrEP counseling when introducing new options. Opinions on the vaginal ring were divided—some women valued its discretion and ease of use, while others were uncomfortable with the concept of self-insertion. Additionally, participants noted that clinic visits for their baby could serve as a convenient way to discreetly attend follow-up appointments for injectable PrEP. This study offers valuable insights to support the integration of LA-PrEP methods into antenatal care (ANC) settings and identifies strategies to promote their acceptability and adoption among this population.

In contrast to previous literature, women in this study more often expressed an aversion to the vaginal ring. Previous studies conducted in South Africa, Malawi, Zimbabwe, and Uganda on DVR acceptability stand in contrast to the view expressed by women in this study. These studies found that both pregnant and breastfeeding participants with experience using the DVR considered it acceptable, easy to use, user friendly, and minimally burdensome [24–26]. Participants of this study only had experience with daily oral PrEP, not with DVR, but they often expressed an aversion to the vaginal ring. This notable difference in DVR experience and acceptability highlights that barriers to acceptability for this option may be overcome with appropriate education, counselling, and support. Future studies should explore offering all available forms of PrEP to postpartum women to better understand their acceptability and preferences for different options within the local context.

There were several themes of acceptability that were unique to pregnancy and breastfeeding periods. Women mentioned that safety for their baby while pregnant was a core driver of their acceptability of any PrEP option and that this concern was alleviated in the postpartum period. However, they indicated that counseling or information from healthcare providers about safety data could enhance their confidence in its use during pregnancy. Participants further noted that if the safety of these options was well established, they would be open to using LA-PrEP during pregnancy. These components of acceptability are in line with previous literature around the importance pregnant and breastfeeding women place on safety for their baby [9, 26, 27]. As more PrEP options become available for this population, it is crucial to address concerns and knowledge gaps about how active agents may affect the baby, while also recognizing the strong motivations of pregnant women to keep their baby HIV-free [11].

Women in this study identified several strategies that were important to support acceptance of different PrEP methods. Most often identified was education from healthcare workers on availability of PrEP options, side effects, safety, and effectiveness. Safety for their baby was especially important for pregnant and breastfeeding mothers and often the driver of their acceptance for different PrEP choices. Including pregnant and breastfeeding women in PrEP clinical trials and bolstering safety profiles of new PrEP options is imperative to ensure acceptability in this important population [28]. Additional strategies such as video-based PrEP information in the waiting bay [29] and decision support tools [30] have been shown to improve PrEP knowledge and persistence and can be adapted for LA-PrEP options as well. Elements of acceptability elucidated in this study can be used to adapt existing PrEP support tools for pregnant and postpartum women.

### Limitations

This study has several limitations that should be considered when interpreting the findings. First, the eligibility criteria for the clinical trial focused exclusively on women with high empiric HIV risk scores who initiated daily oral PrEP in pregnancy, yet most women who participated in these IDIs were over a year postpartum. As a result, there is a potential for recall bias when participants were asked to reflect on the acceptability of interventions during pregnancy which limits the applicability of the findings to women currently experiencing pregnancy. Second, social desirability bias may have influenced participants’ responses, particularly when discussing sensitive topics such as HIV prevention and intervention strategies. Participants may have been inclined to provide responses they believed were expected or socially acceptable rather than fully sharing their personal experiences or opinions. Lastly, the identified strategies to support acceptability were primarily centered around SMS-based support tools, which all participants received as part of their enrollment in the clinical trial. This focus may have limited participants’ consideration of alternative strategies or innovative ideas outside the SMS system, potentially constraining the breadth of the feedback and suggestions provided. Despite these limitations, the study provides valuable insights into the perspectives of women at high risk for HIV and highlights important considerations for future intervention design and implementation.

## Conclusion

This qualitative study offers valuable insights into the acceptability of different HIV PrEP options among postpartum women with experience taking PrEP during pregnancy, a population often underrepresented in HIV prevention research. The findings reveal diverse preferences and highlight key factors influencing acceptability, including safety, discretion, and convenience. While long-acting options, particularly injectable PrEP, were broadly accepted, the vaginal ring’s acceptability was more variable, emphasizing the importance of tailored education and support. The study also underscores the distinct needs of pregnant versus postpartum women, particularly their concern for their baby’s safety during pregnancy. Addressing knowledge gaps and enhancing support strategies, including healthcare worker education and innovative tools, are important next steps for improving PrEP uptake and persistence among pregnant and postpartum women at high risk for HIV.

## Figures and Tables

**Table 1:** Operationalized definitions of the Theoretical Framework of Acceptability (TFA) constructs

TFA construct	Operationalized definition
Affective attitude	Postpartum women’s feelings about using different PrEP methods
Perceived effectiveness	The extent to which different PrEP methods are perceived by postpartum women as likely to prevent HIV
Ethicality	The extent to which different PrEP methods have a good fit with postpartum women’s value system
Intervention Coherence	The extent to which postpartum women understand different PrEP methods and how they work
Opportunity Costs	The extent to which benefits, profits, or values must be given up by postpartum women to use different PrEP methods
Self-Efficacy	Postpartum women’s confidence that they can perform the behavior(s) required to use different PrEP methods

**Table 2:** Sociodemographic characteristics of IDI participants (n = 70)

	N = 70^1^
Age (years)	25.0 (22.0, 30.5)
Currently married	49 (70.0%)
High ACE score	16 (22.9%)
Secondary education or higher	46 (65.7%)
Has regular employment	13 (18.6%)
2 or more people per room	13 (18.6%)
Partner HIV status	
HIV negative	7 (10.0%)
Unknown status	60 (85.7%)
No partner	3 (4.3%)
Previous number of pregnancies	2.0 (1.0, 3.0)
Primigravida	26 (37.1%)
Previous pregnancy loss	7 (10.0%)
Discontinued PrEP in pregnancy	7 (10.0%)
Discontinued PrEP in postpartum	25 (35.7%)
Used FP by 9 months postpartum	51 (72.9%)
Used injectable FP by 9 months postpartum	20 (28.6%)

1 n (%); Median (IQR)

ACE: Adverse childhood experience; FP: Family planning

## Data Availability

The datasets generated and/or analyzed during the current study are not publicly available due to institutional data sharing policies and restrictions but may available from the corresponding author on reasonable request.
